# Low-cost lipid production by an oleaginous yeast cultured in non-sterile conditions using model waste resources

**DOI:** 10.1186/1754-6834-7-34

**Published:** 2014-03-04

**Authors:** Fabio Santamauro, Fraeya M Whiffin, Rod J Scott, Christopher J Chuck

**Affiliations:** 1 Department of Biology and Biochemistry, University of Bath, Bath BA2 7AY, UK; 2 Centre for Sustainable Chemical Technologies, Department of Chemical Engineering, University of Bath, Bath BA2 7AY, UK

**Keywords:** Biodiesel, Lipid, Yeast, Heterotrophic, Waste, Pilot scale, Glycerol, Lignocellulose

## Abstract

**Background:**

The yeast *Metschnikowia pulcherrima*, previously utilised as a biological control agent, was evaluated for its potential to produce lipids for biofuel production.

**Results:**

Cultivation in low cost non-sterile conditions was achieved by exploiting its ability to grow at low temperature and pH and to produce natural antimicrobial compounds. Although not previously classified as oleaginous, a combination of low temperature and restricted nutrient availability triggered high levels of oil production in *M. pulcherrima* cultures. This regime was designed to trigger the sporulation process but prevent its completion to allow the accumulation of a subset of a normally transitional, but oil-rich, ‘pulcherrima’ cell type. This approach resulted in yields of up to 40% lipid, which compares favourably with other oleaginous microbes. We also demonstrate that *M. pulcherrima* metabolises glycerol and a diverse range of other sugars, suggesting that heterogeneous biomass could provide a suitable carbon source. *M. pulcherrima* also grows well in a minimal media containing no yeast extract. Finally, we demonstrate the potential of the yeast to produce lipids inexpensively on an industrial scale by culturing the yeast in a 500 L, open air, tank reactor without any significant contamination.

**Conclusions:**

The production of antimicrobial compounds coupled to efficient growth at low temperature and pH enables culture of this oleaginous yeast in inexpensive, non-sterile conditions providing a potential route to economic biofuel production.

## Background

The rising cost of fossil fuels, coupled to concerns over security of supply and an increasing awareness of the environmental impact of associated CO_2_ emissions, have led to the development of alternative energy solutions becoming a global priority. Some of the most ambitious national targets have been set by the United Kingdom, where the government is committed to sourcing 15% of the total energy demand from renewable resources by 2020, and 80% by 2050 [1]. Since a quarter of emissions are produced by the transport sector, meeting these reduction targets requires urgent development of biofuels compatible with the current infrastructure. Currently, the main source of biofuels is sugar-derived bioethanol or biodiesel produced from lipids, derived from oil palm, rapeseed or soybean [2]. However, these crops compete with food crops for agricultural land and consequently have both a negative public image and environmental impact [3]. Oleaginous microorganisms such as microalgae, bacteria, fungi and yeasts are an attractive alternative to higher plants for lipid production, since their production does not require agricultural land, avoiding displacement of food production. While species from these taxa have the potential to produce lipids, only microalgae have been extensively investigated to date [4]. Although algae have great theoretical potential as a fuel source, substantial technical hurdles currently prevent their cost-effective exploitation. These include the scarcity of suitable land, given the current levels of productivity, the lack of adequate photosynthetically active radiation and the prohibitive costs of delivering supplementary light, fertiliser, controlling temperature and critically, protecting against invasive non-lipid-producing species [5,6].

Heterotrophic organisms, such as yeasts, offer a credible alternative to microalgae for the production of biofuel feedstocks, especially in Northern Europe. Oleaginous yeast species are highly productive on a per-cell basis, with lipid yields of up to 65% dry weight and grow to high densities with biomass yields of 10 to 100 g⋅L^-1^ reported over 3 to 7 days [7]. In comparison, microalgae achieve only 0.15 to 0.25 g⋅L^-1^ per day in open pond systems [5]. The most common yeasts so far examined for biofuel production are *Rhodotorula glutinis*, *Yarrowia lipolytica* and *Lipomyces starkeyi*[8-11].

Recently, the economic production of C_5_ and C_6_ sugars from waste cellulosic materials has become plausible, making plant sugars derived from lignocellulose a feasible source of renewable feedstocks [12,13]. Unlike microalgae, yeast cultivation does not require light, which both reduces input costs and enables production 24 h per day. Essential inputs such as phosphorous and nitrogen are also available from waste streams such as waste water, again reducing production costs.

One key issue with the cultivation of heterotrophic organisms is maintaining an axenic culture. Bacteria from the surrounding environment or contaminating input waste streams have the potential to outcompete most types of oleaginous yeast. Ensuring the strict sterile conditions required for production of triglycerides by yeast, therefore, requires stringent pre-treatment of the feedstock and careful enclosure of the fermentation broth. Both significantly increase production costs, potentially limiting industrial-scale lipid biofuel production [14]. One potential solution is to use an extremophile yeast that thrives at an extreme pH, temperature or salinity that significantly reduces or prevents competing microbial growth [15,16]. As bacteria and other contaminating organisms can adapt to such environments, an extremophile with more than one protective mechanism is attractive for open-tank reactors.

The non*-*saccharomyces yeast, *Metschnikowia pulcherrima*, is known to severely limit the growth of other microbes in the natural environment and is consequently used commercially as a post-harvest control agent [17]. However, the ecological significance and exact mechanisms are still poorly understood but offer the potential for commercial exploitation. *M. pulcherrima*, also reported in literature as *Candida pulcherrima* (anamorph), *Saccharomyces pulcherrimus*, *Rhodotorula pulcherrima*, *Torula pulcherrima*, *Cryptococcus castellanii* and *Torulopsis pulcherrima*, [18] is commonly found in flower nectar and fruit surfaces, and colonises the grape must after pressing, contributing to the flavour and aroma of the wine [19]. The yeast thrives in the must despite its high acidity (pH 3 to 4) and sugar concentration (>100 g/L), and resists competition from other microorganisms. Cultures of *M. pulcherrima* do not require iron or vitamins other than biotin [20,21]. *M. pulcherrima* is well-known for its production of the red pigment, pulcherrimin formed by irreversible binding of pulcherrimic acid and environmental iron to create a metal-organic framework. This sequestration prevents competing microbes accessing essential supplies of iron, and is the assumed basis of the use of *M. pulcherrima* as a biofungicide in post-harvest disease control [17,22,23]. *M. pulcherrima* was therefore selected for study for its potential for low-cost culture in non-sterile conditions due a combination of acidophilia and secretion of the potent anti-microbial compounds, pulcherriminic acid and 2-phenylethanol [24].

During its growth on the surface of grapes *M. pulcherrima* is assumed to catabolise constituents of the plant cell-wall, as well as accessing sugars from within the grape [25]. This accords with recent studies demonstrating that *M. pulcherrima* produces a wide range of cell-wall degrading enzymes; this property indicates a degree of metabolic plasticity that lends itself to industrial applications in oenology [19,24].

Whilst the innate ability of *M. pulcherrima* to grow at low pH and temperature combined with pulcherrimin production would facilitate its culture under relatively inexpensive non-sterile conditions, previous studies have concluded that the species is not oleaginous [26]. However, when subjected to environmental stress, for example, nitrogen starvation, a subset of cells within an *M. pulcherrima* culture begin sporulation via a transitional pulcherrima cell-type that has shown some evidence of lipid accumulation [27,28]. We reasoned that increasing the proportion of pulcherrima cells formed in response to a suitable stimulus, coupled with a means to prevent their development into oil-poor spores, could elevate overall lipid production by an *M. pulcherrima* culture into the oleaginous range.

Here we show that manipulating the culture regime successfully increased the proportion of oil-rich pulcherrima cells and achieved a 40% overall lipid yield. We also show that the yeast metabolises glycerol and a diverse range of other sugars, suggesting that heterogeneous biomass could provide a suitable carbon source. Finally, we illustrate the potential to produce lipids inexpensively on an industrial scale by culturing *M. pulcherrima* in a 500-L, open air, tank reactor.

## Results and discussion

As a non-saccharomyces yeast involved in the first stages of wine fermentation, *M. pulcherrima* endures particularly stressful conditions in the grape must, such as high acidity due to the high levels of tartaric and malic acid, and high osmotic pressure due to the high sugar concentrations. The ability to thrive under such harsh conditions makes *M. pulcherrima* a potentially attractive organism for industrial applications, particularly where culture in non-sterile conditions would reduce production costs. Whilst the robustness of *M. pulcherrima* has been established, its status as an oleaginous yeast has not. We set out to determine whether *M. pulcherrima* is capable of high lipid-production in conditions that exploit its potential for low cost culture.

### Triggering oil production in *M. pulcherrima*

Lipid accumulation in microbial cells normally requires a starvation step to promote energy storage at the expense of further cell division [29]. To achieve this, the starting concentration of nitrogen in the culture medium is deliberately limited relative to the carbon source. A recent study evaluating the potential of yeasts to produce biofuels from glycerol found that *M. pulcherrima* did not accumulate lipids to any significant degree at 28°C, over 5 days, even at high C/N ratios [26]. However, other types of stress induce *M. pulcherrima* to undergo sporulation via a transitory pulcherrima cell-type that accumulates high lipid levels, particularly in dilute media or at low temperatures, though the effectiveness of these treatments has not been quantified [27,28]. Although pulcherrima cells have not been observed as the predominant cell-type in a culture, it seemed likely that conditions of nutrient starvation and below-ambient temperatures would act as a trigger for this stage. Such low temperatures would potentially halt sporulation, resulting in increased levels of lipid-containing cells. To determine whether temperature could trigger oil production in *M. pulcherrima,* the culture was grown at 20°C for 3 days, the temperature was then modified (increased or decreased) for the remaining length of the culture period. The lipid content of the biomass was measured for the cultures after 15 days.

**Figure 1:**
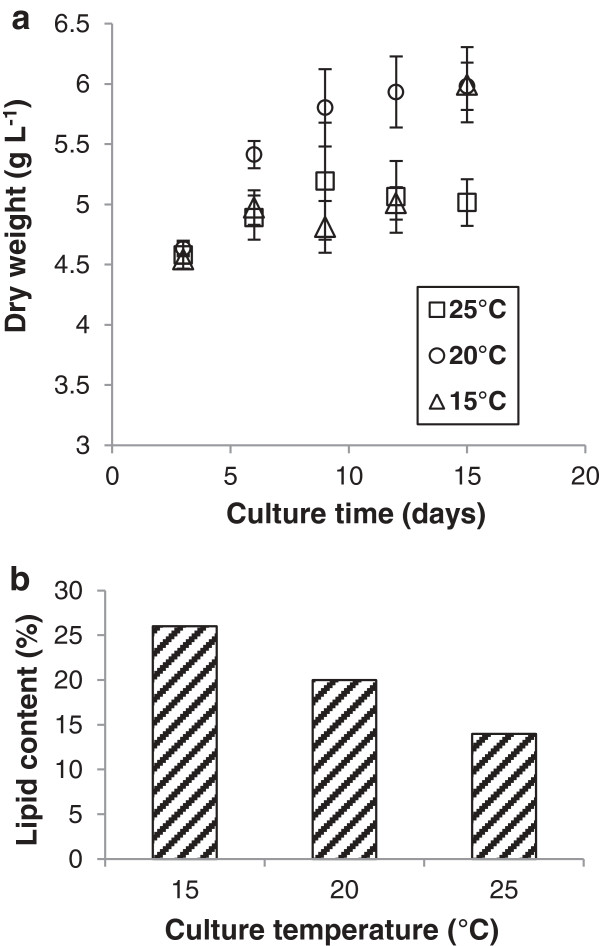
**Effect of temperature on the growth of *M. pulcherrima *** **grown on standard *M. pulcherrima *** **(SMP) media (a) and total lipid content as a percentage of dry weight after 15 days growth (b).**

At 25°C, 14% lipid was extracted from the cultures, which is approaching the oleaginous range (Figure 1). As predicted, the highest lipid productivity was observed at 15°C, with per-cell lipid content of 26%. Although we did not attempt to quantify the proportion of pulcherrima in these cultures, the dramatic increase in overall lipid content suggests a substantial enrichment had been achieved for the reason outlined above. Significantly, *M. pulcherrima* grew extremely well between 15°C and 20°C, reaching values of dry biomass around 6 g/L. When the stationary phase was held at 25°C a 20% reduction in dry biomass was observed, though there was little difference in the lipid production of the system compared to 20°C. Not only are these temperatures too low for most common bacteria but are also ideal for producing fuels in Northern Europe without high energy-costs for heating.

### The effect of restricted nutrients on the growth

**Figure 2:**
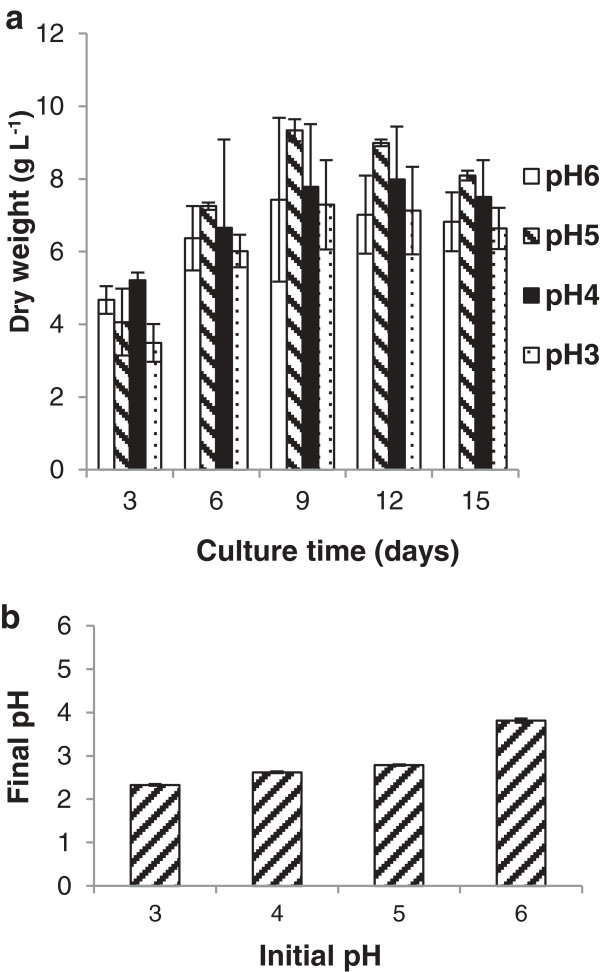
**Effect of the initial pH on the growth of the *M. pulcherrima *** **culture grown at 20°C over 15 days (a) and the final pH of the cultures (b).**

One of the main factors in maintaining a monoculture is the ability of *M. pulcherrima* to grow at low pH. In the literature, *M. pulcherrima* is reported to grow optimally at pH between 5.0 and 7.5, but has been shown to grow at pH as low as 3.0 [22,30]. Under the conditions used in this study *M. pulcherrima* grew well between pH 3.0 and pH 6.0, though the maximum biomass was observed at an initial pH of 5.0 (Figure 2a). To ensure that the conditions were capable of supporting significant lipid accumulation, a semi-quantitative assay was used. For this samples were collected at regular intervals, stained with the fluorescent dye Bodipy 493/503 and analysed by flow cytometry. Biomass production was only reduced slightly at pH 4.0. Even at pH 3.0, around 85% of the biomass was produced compared to that at pH 5.0. Irrespective of the starting pH, or change in pH during the culture period, biomass productivity remained above 5 g/L. Irrespective of the starting pH, *M. pulcherrima* acidified the environment, resulting in a pH slightly above 2 (Figure 2b). While presumably too low for optimal biomass production, this would further insure that the cultures remain sterile and that no bacteria could outcompete the yeast.

**Figure 3:**
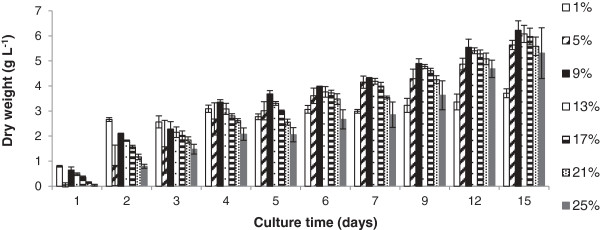
**Effect of glycerol concentration on the growth of a culture of *M. pulcherrima*, grown at 20°C over 15 days with a starting pH of 5.**

The original screening, to promote lipid accumulation in *M. pulcherrima* utilised glycerol as a feedstock [26]. Glycerol is an important waste product derived from the biodiesel production process as an aqueous solution (approximately 65% wt/wt) contaminated with small amounts (below 5% wt/wt of each contaminant) of compounds such as methanol, catalyst and organic compounds. These contaminants have not been found to significantly affect the growth of other microorganisms compared to pure glycerol [31]. We therefore first tested the ability of *M. pulcherrima* to exclusively utilize 95% pure glycerol, a model waste resource, across a broad range of concentrations (1 to 25%). The biomass content increased steadily over the 15-day culture period irrespective of the glycerol concentration (Figure 3). The maximum biomass yield was achieved with a 9% glycerol concentration, which decreased only slightly at higher concentrations. Although we did not investigate the cause of this reduction, high glycerol content could inhibit oxygen uptake or create high osmotic pressures sufficient to inhibit culture growth. However, this effect was minor and our data therefore demonstrate that the yeast thrives on a wide range of glycerol concentrations. Significantly, biomass productivity was above 5 g/L and constant at glycerol concentrations between 5 and 9%, indicating that there is little benefit in biomass terms from almost doubling the glycerol concentration.

Several other *Candida* species *(Candida curvata, Rhodotorula graminis, Cryptococcus curvatus* and *Yarrowia lipolytica)* reportedly produce between 28 and 53% lipid when grown on glycerol as a carbon source. This level is comparable to that produced by the *M. pulcherrima* strain presented in this work [32,33]. *M. pulcherrima* grew at high glycerol concentrations, and although this has been reported for some oleaginous species such as *Y. lipolytica*, it is far from common. For example, other similar oleaginous species, such as *C. curvatus* are unable to grow on concentrations higher than 60 g/L, presumably due to the elevated osmotic pressure [31-33].

**Figure 4:**
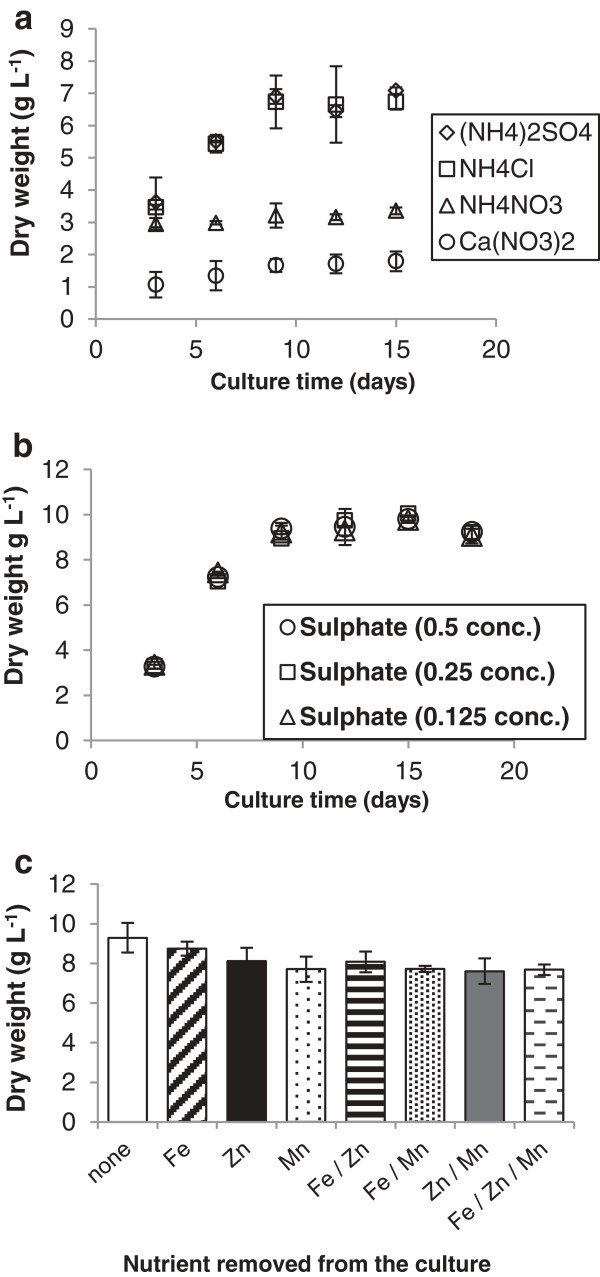
**Nutrient requirements for *M. pulcherrima *cultivation on glycerol. a)** Nitrogen source, **b)** sulphur content and **c)** demonstrates the removal of micronutrients.

A key issue when using waste feedstocks is the potential heterogeneity of supply, which demands that the cultured organism possesses a degree of flexibility in its nutrient and environmental requirements. To assess *M. pulcherrima* for its potential for robust waste feedstock utilisation we measured the impact of different nutrient sources on growth dynamics. Three alternative nitrogen sources (NH_4_Cl, NH_4_NO_3_ and Ca(NO_3_)_2_) were evaluated by replacing (NH_4_)_2_SO_4_ in the standard *M. pulcherrima* (SMP) containing 10% w/v glycerol. The total nitrogen concentration was kept constant when using the different nitrogen sources. Biomass productivity was greatest using ammonium salts compared to nitrates (Figure 4a), with NH_4_Cl supporting a growth rate comparable to (NH_4_)_2_SO_4._ In contrast, the performance of NH_4_NO_3_ and Ca(NO_3_)_2_ was below 3 g/L indicating that these compounds are not suitable nitrogen sources for *M. pulcherrima.*

Sulphur is another key nutrient that can affect growth rates. Generally sulphur is present as sulphates, especially in waste water. To assess the flexibility of *M. pulcherrima*, a range of cultures were examined with reduced levels of sulphate (Figure 4b). There was no difference, in terms of dry biomass; on culturing *M. pulcherrima* with concentrations of sulphur between 0.321 g/L and 0.045 g/L, the same level of biomass were obtained.

A range of micronutrients is added to most yeast culture media, including salts of manganese, zinc and iron. Although these micronutrients are potentially present in waste water, the type and amount of the micronutrient will vary substantially [34]. To determine the tolerance of *M. pulcherrima* to fluctuations in micronutrient availability, the yeast was grown in SMP (10% w/v glycerol) lacking Fe, Zn, or Mn, either singly or in combination. The biomass production after 15 days was then compared to a culture grown on SMP with all the nutrients present (Figure 4c). Surprisingly, biomass productivity was reduced by only 15% in SMP not supplemented with iron, zinc and manganese.

*M. pulcherrima* demonstrates excellent flexibility on being cultured with different types and amounts of nitrogen, sulphur and micronutrients. This demonstrates that although there is an optimal culture configuration that produces the highest levels of biomass from any given system, the effect of removing key nutrients does not dramatically reduce biomass yields. *M. pulcherrima*, therefore, has excellent adaptability to any changes in the waste water or alternative nutrient streams that could be used to culture the system.

**Figure 5:**
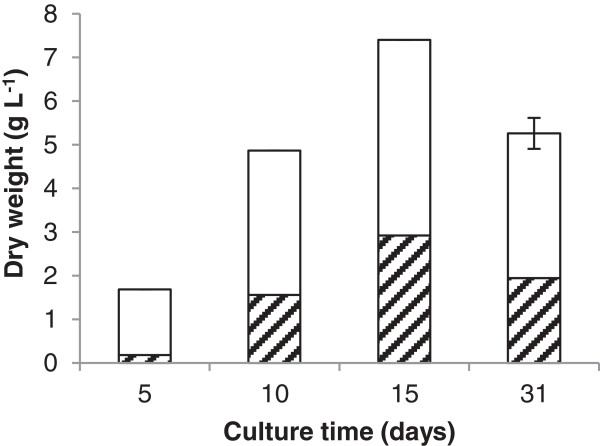
Total biomass and lipid produced (shaded area) using an optimised media.

Following these promising results we modified SMP to optimise lipid accumulation. The new medium, optimised *M. pulcherrima* (OMP) medium lacked manganese and iron, and had lower levels of sulphur. Cultures grown in this medium achieved higher lipid content whilst retaining biomass yields of around 7 g/L. In a typical experiment *M. pulcherrima* was grown in OMP at 25°C for the first 3 days, than at 15°C for a further 12 days. After 15 days a 7.4 g⋅L^-1^ biomass yield was achieved, which had a total lipid content of 40% (Figure 5). This demonstrates that with an appropriate combination of medium and temperature regime, the oil productivity of *M. pulcherrima* is well into the oleaginous range.

Lipid accumulation in *M. pulcherrima* continues up to 15 days, after which the lipid content decreases, presumably due to cellular lipid turnover (Figure 5). This phenomenon has been reported for the yeast *Y. lipolytica* and other oleaginous fungi, for example, *Cunninghamella echinulata*[35]. The authors propose that the lipid degradation occurs in *C. echinulata* by selective use of triglyceride to produce glycolipids, sphingolipids and phospholipids through the substitution of one or more fatty acid (FA) chains. The triglyceride can also be completely hydrolysed to glycerol and free fatty acids, which can then be funnelled into glycolysis and β-oxidation.

### Alternative feedstocks

**Figure 6:**
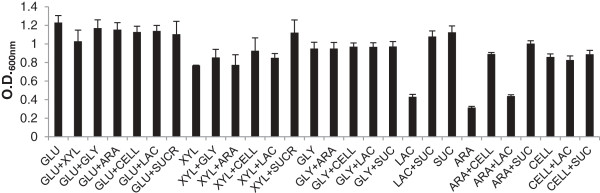
**Biomass productivity of *M. pulcherrima *on a range of simple sugars.** Optical density (O.D.) of the culture after 15 days. GLU, glucose; XYL, xylose; GLY, glycerol; ARA, arabinose; CELL, cellobiose; LAC, lactose; SUC, sucrose.

Although *M. pulcherrima* is productive using a glycerol carbon source, a far more abundant feedstock is lignocellulose. Waste food, agricultural wastes or energy crops grown specifically for the purpose can be converted into a range of sugars through relatively inexpensive chemical or enzymatic processes [36]. The composition of the sugars obtained depends heavily on the method and source of the feedstock, and the main sugars are glucose, arabinose, xylose and cellobiose, and a range of oligosaccharides [37]. To establish its ability to utilise depolymerised lignocellulose, *M. pulcherrima* was cultivated on a wide range of sugars including lactose and sucrose (Figure 6). Biomass productivity for *M. pulcherrima* was higher in media containing glucose compared to any other sugar or combination of sugars. Provided glucose was present, any other sugar acted as an effective carbon source with little reduction in biomass yield. A possible explanation is that glucose induces *M. pulcherrima* to express enzymes capable of metabolising oligosaccharides, as occurs in a range of non-saccharomyces yeast [38]. Although *M. pulcherrima* was capable of growth on xylose, this resulted in reduced biomass productivity compared to glucose. *M. pulcherrima* struggles to grow on the arabinose aldehyde and lactose but metabolises sucrose extremely effectively, obtaining dry biomass content similar to glucose. Overall, *M. pulcherrima* can be cultivated effectively on a range of sugars, especially in the presence of glucose, indicating its suitability to utilize abundant biomass sources, such as lignocellulose.

**Figure 7:**
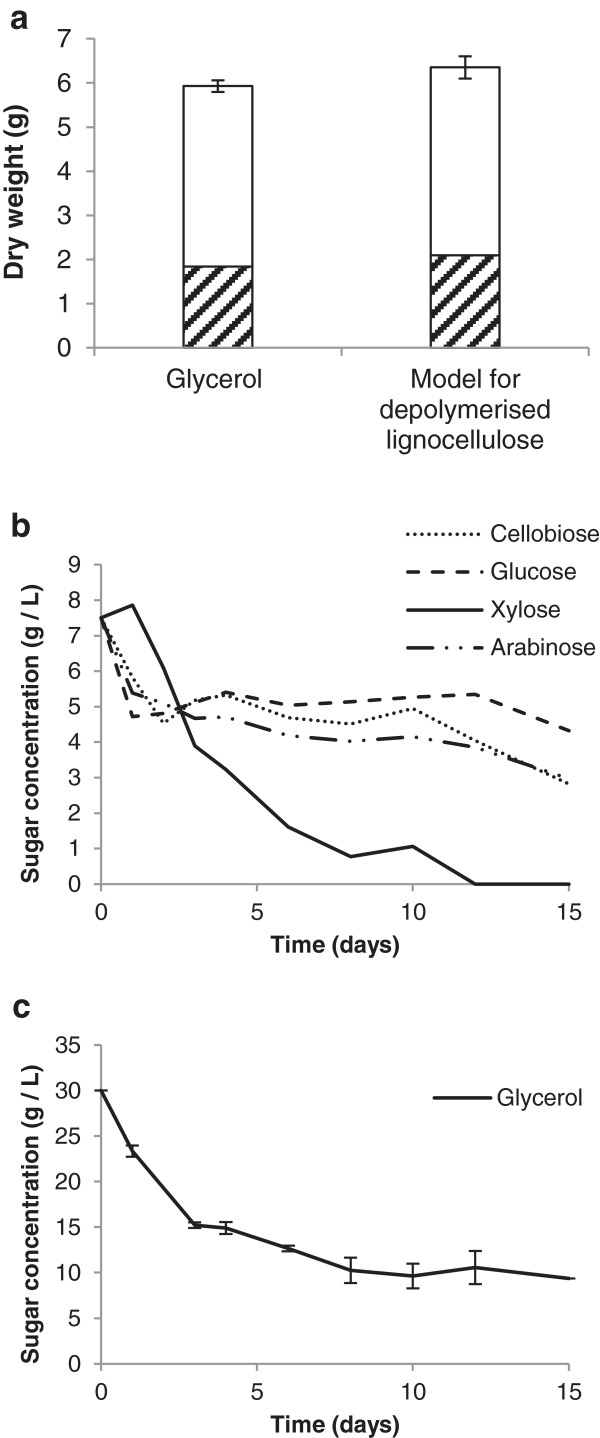
**Total biomass and lipid produced (shaded area) for glycerol and a model for depolymerised lignocellulose in optimised media, and consumption of sugars and glycerol over the culture period. a)** Total biomass and lipid produced and model for depolymerised lignocelluloses. The model contained 7.5 g, arabinose, 7.5 g xylose, 7.5 g glucose and 7.5 g cellobiose as the carbon source. **b)** Consumption of sugars. **c)** The consumption of glycerol over the culture period.

As the main products of the depolymerisation of lignocellulose are glucose, arabinose, xylose and cellobiose, *M. pulcherrima* was cultured on a mixture of these sugars as a model waste resource and the biomass and lipid productivity established (Figure 7). *M. pulcherrima* produced amounts of biomass and lipid on this feedstock similar to levels achieved on glycerol. Significantly, with the exception of xylose, all four sugars of the model feedstock were starting to be catabolised by *M. pulcherrima* within the first 24 hours. Xylose consumption commenced within 48 hours. By 72 hours the culture had entered the stationary phase (data not shown), and the consumption of glucose, arabinose and cellobiose practically halted. Interestingly, xylose was continually consumed until exhausted after 12 days. It is possible that *M. pulcherrima* catabolises xylose directly, or that xylose is converted into an unknown secondary product. On exhaustion of the xylose, *M. pulcherrima* resumed using other sugars in the broth. After 15 days, both the glycerol and model lignocellulose cultures, had consumed nearly 20 g⋅L^-1^ of sugar, which was two thirds of the original starting culture.

**Figure 8:**
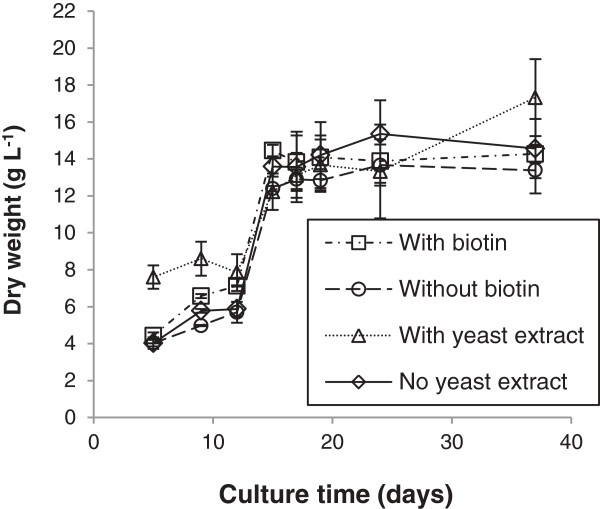
**Fed-batch culture of *M. pulcherrima a *** **with additional nutrients added every 2 days until 24 days, then kept at 15°C for 12 days.**

To increase the cell density, separate cultures were investigated under a fed-batch regime, using equal amounts of the four sugars and varying the amount of yeast extract, biotin and additional nitrogen (Table 1, Figure 8). To verify whether *M. pulcherrima* was capable of contamination-free growth in a fed-batch system the yeast was cultured in media made up in final effluent waste water. None of the reagents were sterilised prior to use. Each culture was fed every 2 to 3 days up to 24 days (25°C, 180 rpm) and then allowed to rest at 15°C for 12 days without agitation. Other than yeast extract, biotin and the carbon source all nutrients were supplied according to the OMP media, except phosphate, which was supplemented only at 15 days since the starting concentration high excess compared to the nutritional requirement on the yeast.

**Table 1:** **Lipid profile of fed-batch cultures of *M. pulcherrima***

	**Run 1 (with yeast extract)**	**Run 2 (without yeast extract)**	**Run 3 (with biotin)**	**Run 4 (without biotin)**
**Yeast extract**	+	-	-	-
**Biotin**	-	+	+	-
**Additional NH4Cl**	-	+	-	-
**Final biomass yield (g⋅L**^**-1 **^**)**	17.3	14.6	14.3	13.4
**Final lipid content (% dry weight)**	35	39	19	33

**+ =** nutrient included, - = nutrient absent.

*M. pulcherrima* was successfully grown in a fed-batch culture, with over 17 g⋅L^-1^ biomass being produced over 30 days. Unsurprisingly, the highest biomass yields were achieved with yeast extract, though this is not essential to growth as comparable productivity was observed for all cultures irrespective of the supplementary nutrients. The highest lipid content of 39%, was produced by the culture without yeast extract but with a supplementation of biotin and nitrogen. Surprisingly, dry biomass productivity around 13 g/L and lipid productivity around 33% were still observed in the cultures containing no yeast extract, biotin or additional nitrogen. The ability to produce lipids from a complex sugar feed, with no additional yeast extract or vitamin supplementation, has significant ramifications for the viability of any future industrial process.

### Open-air stirred tank production

**Figure 9:**
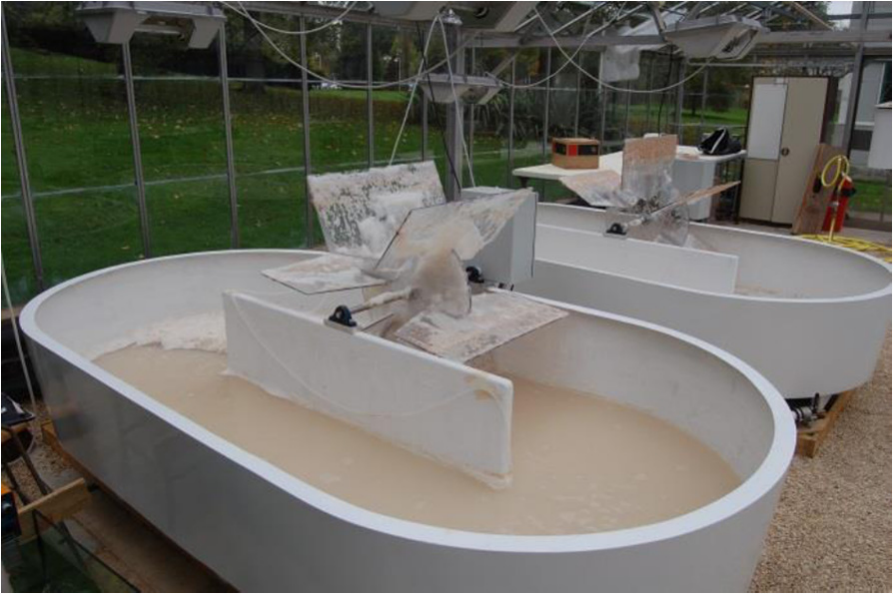
**Raceway pond cultures of *M. pulcherrima*.**

**Figure 10:**
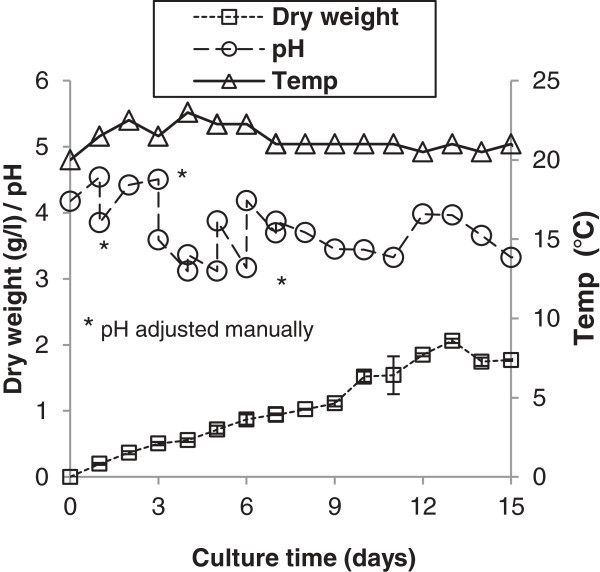
**Temperature, pH and growth curve for the *M. pulcherrima *** **culture, cultivated in an open-air stirred tank reactor.**

To demonstrate the suitability of *M. pulcherrima* for low-cost production, it was grown in a hostile environment with limited temperature control on a model waste resource. The yeast was cultured in a glycerol-based OMP media in non-sterile conditions in two 500-L raceway ponds situated in a temperature-controlled glasshouse (Figure 9). The ponds were gently agitated by a paddle wheel at 10 rpm and maintained for 15 days (Figure 10). The temperature remained roughly constant at 21°C irrespective of the conditions outside the glasshouse. During culture, *M. pulcherrima* was expected to regulate the pH of the medium by producing both acids and bases depending on the stage of the growth cycle. To maintain a healthy population of *M. pulcherrima*, while retaining acceptable rates of lipid and biomass production, pH was artificially held between 3 and 4 by the addition of weak solutions of either HCl or KOH. Although some bacteria were observed at various times over the first 72 hours of culture, the population remained overwhelmingly *M. pulcherrima* (as judged by flow cytometry; data not shown). After 2 weeks when the pH was manually reduced to 3, no further contamination was observed for the remainder of the culture period. When the cultures in the raceway ponds reached a value of absorbance at 600 nm of around 10, many large *M. pulcherrima* colonies were observed growing on the paddle wheels. These colonies developed an intense pink colour compared to the suspended culture, presumably due to increased pulcherrimin production associated with elevated levels of oxygenation [20]. Over the culture period, biomass accumulation measured by flow cytometry increased steadily reaching a final value of 2.06 g⋅L^-1^ (Table 2).

**Table 2:** **Biomass characteristics and lipid profile of the oil produced from *M. pulcherrima *** **grown in the raceway ponds**

**Component**		**Value**
**Biomass**		2.06 g⋅L^-1^
**Lipid**		34 wt%
**Sterol**		18 mol%
**Fatty acids**	**14:0**	<1%
	**15:0**	<1%
	**16:0**	21%
	**16:1**	8%
	**17:0**	1%
	**18:0**	4%
	**18:1**	50%
	**18:2**	11%
	**18:3**	7%
	**19:0**	<1%
	**21:0**	<1%

Although total biomass yield was lower than obtained at laboratory scale, these suboptimal conditions nevertheless outperformed similar systems used to culture microalgae [39]. Alongside sterilisation, agitation is a major cost in running an industrial bioprocess [13]. Though further work is needed to optimise cultures of *M. pulcherrima* on this scale, the preliminary findings demonstrate that the yeast can be cultured under non-sterile conditions, with minimal agitation. This has the potential to substantially reduce the costs of a process. The level of oil extracted from the harvested biomass was 34% of the dry weight.

## Conclusions

The biological control yeast, *M. pulcherrima,* which was previously classified as non-oleaginous, is shown here capable of producing up to 40% lipid by manipulating pH and temperature. The yeast also uses a variety of sugars potentially sourced from waste lignocellulose. Fed-batch culture using these sugars achieved a total biomass yield of 17 g⋅L^-1^. High biomass productivity was also achieved using simplified media lacking expensive inputs such as yeast extract and biotin. The unique combination of acidophilia and production of antimicrobials enable culture in lost-cost non-sterile conditions providing a potentially economically viable biofuel production platform.

## Methods

### Materials

All chemicals were purchased from Sigma Aldrich unless otherwise stated and used without purification.

### Culture conditions on a laboratory scale

Cultures were grown from a single colony taken from a yeast extract, malt extract and dextrose (YMD) agar plate suspended in 10 ml YMD (yeast extract 10 g⋅L^-1^; malt extract 20 g⋅L^-1^; glucose 20 g⋅L^-1^) and inoculated into liquid standard *M. pulcherrima* (SMP) medium adapted from Chatzifragkou *et al*. (KH_2_PO_4_ 7 g⋅L^-1^; Na_2_HPO_4_ 2.5 g⋅L^-1^; MgSO_4_^.^ 7H_2_O 1.5 g⋅L^-1^; CaCl_2_^.^ 2 H_2_O 0.15 g⋅L^-1^; ZnSO_4_^.^ 7H_2_O 0.02 g⋅L^-1^; MnSO4 ^.^ H_2_O 0.06 g⋅L^-1^; FeCl_3_ 0.15 g⋅L^-1^; (NH_4_)_2_SO_4_ 0.5 g⋅L^-1^ and yeast extract 1 g⋅L^-1^) [23]. The medium was autoclaved for 20 minutes at 120°C prior to use. Each experiment was conducted in triplicate using 10 ml SMP medium in 50-ml Falcon tubes.

The optimum glycerol concentration was determined using SMP medium containing between 1% and 25% (w/v) glycerol. To examine the effect of temperature on lipid production, cultures were grown for 3 days at 25°C then switched to either 15°C or 20°C for the remaining period. Alternative nitrogen sources were evaluated by replacing (NH_4_)_2_SO_4_ in SMP medium (10% w/v glycerol) and maintaining the total nitrogen concentration at 0.106 g⋅L^-1^. Micronutrient deficiency was investigated using SMP medium (10% w/v glycerol) lacking the specified micronutrient. Sulphur content was varied using modified SMP medium (10% w/v glycerol) containing 50%, 25% and 12.5% of the concentration in SMP media. pH was adjusted using dilute HCl or KOH to a achieve a range between pH 3 and pH 6. Each set of experiments were conducted over 15 days. Optical density (O.D.)_600nm_, cell number and lipid fluorescence were measured at 72-hour intervals.

### Alternative sugar sources

The performance of *M. pulcherrima* on different sugar sources (glucose, glycerol, xylose, arabinose, cellobiose, lactose, sucrose and glycerol) was examined using a 96-well microtitre plate system (Versamax, Molecular devices, UK). Briefly, 200 μl of culture were made up in 96-well plates in SMP medium containing a 30 g⋅L^-1^ of the sugar. After 15 days at 25°C, 180 rpm, the O.D._600nm_ was measured using a plate reader. All the possible combinations of two sugars were tested using six repeats for each combination. An OMP medium was developed for low-cost culture of *M. pulcherrima*. OMP consisted of: KH_2_PO_4_ 7 g⋅L^-1^; Na_2_HPO_4_ 2.5 g⋅L^-1^; MgSO_4_^.^ 7H_2_O 0.188 g⋅L^-1^; MgCl_2_^.^ 6H_2_O 1.083 g⋅L^-1^; CaCl_2_^.^ 2 H_2_O 0.15 g⋅L^-1^; ZnSO_4_^.^ 7H_2_O 0.02 g⋅L^-1^; (NH_4_)_2_SO_4_ 0.063 g⋅L^-1^; NH_4_Cl 0.405 g⋅L^-1^: yeast extract 1 g⋅L^-1^ and glycerol 30 g⋅L^-1^. OMP was adjusted to pH 5 using dilute HCl and utilised without sterilisation.

*M. pulcherrima* was cultured in a fed-batch regime. The media were comprised of final effluent waste water; all nutrients other than yeast extract, biotin and the carbon source were supplied according to the OMP media. The media were not sterilised prior to use. Each culture was fed every 3 days up to 24 days (25°C, 180 rpm) and then allowed to rest at 15°C for 12 days without agitation, except phosphate, which was supplemented only at 15 days.

### Raceway pond cultivation

OMP with a reduced glycerol content of 30 g⋅L^-1^ was used to establish cultures in two 500-L-capacity raceway ponds situated in a climate-controlled glasshouse. The ponds were inoculated with 500 ml of *M. pulcherrima* culture grown for 48 hours at 25°C with agitation (180 rpm) in yeast extract, mannitol and sorbose (YMS) medium consisting of 30 g L^-1^ yeast extract, 5 g⋅L^-1^ mannitol and 5 g⋅L^-1^ sorbose.

The pond cultures were agitated using a close-fitting paddle wheel driven at 10 rpm and aerated through two spargers situated at opposite sides of the ponds. Culture temperature, pH and O.D._600nm_ were measured daily until the onset of the stationary phase, then every 4 days together with the addition of lipid fluorescence up to 28 days. With the onset of the stationary phase the temperature in the glasshouse was reduced from 25°C to 20°C, the aeration was stopped and the paddle wheels were set at the minimum rotating rate (4 rpm).

### Lipid extraction and analysis

Lipid was extracted by an adaptation of a method first reported by Bligh and Dyer [40]. The samples were stirred in a CHCl_3_ and MeOH mixture (2:1 w/v) over 48 hours, and the biomass was filtered off and washed with additional CHCl_3_. This was repeated three times. The volatiles were removed under reduced pressure and the lipid was analysed.

The lipid and sterol content were calculated by proton nuclear magnetic resonance (^1^H NMR) by comparison of the glyceride protons (δ 4.1 ppm) to the α-protons of the sterol alcohol group (δ 3.7 to 3.9 ppm). NMR spectroscopic measurements were carried out at 298 K using a Bruker AV500 spectrometer (Bruker GmbH, Germany), operating at 300 MHz. ^1^H spectra were typically acquired using a 30-degree excitation pulse and a repetition time of 4.2 sec; 0.3-Hz line broadening was applied before Fourier transform, and spectra were referenced to the residual CHCl_3_ peak from the solvent (*δ* 7.26 ppm).

The glyceride proportion of the oil was transesterified using typical reported techniques [41]. The fatty acid profile was then calculated by gas chromatography-mass spectroscopy (GC-MS) with the resulting fatty acid methyl esters (FAME) component compared to known FAME standards. GC-MS analysis was carried out using an Agilent 7890A gas chromatograph equipped with a capillary column (60 m × 0.250 mm internal diameter) coated with DB-23 ((50%-cyanpropyl)-methylpolysiloxane) stationary phase (0.25-μm film thickness) and an He mobile phase (flow rate: 1.2 ml/minute) coupled with an Agilent 5975C inert MSD with triple axis detector (Agilent Technologies, CA, USA). FAME samples were initially dissolved in 2 ml of dioxane and 1 μl of this solution was loaded onto the column, pre-heated to 150°C. This temperature was held for 5 minutes and then heated to 250°C at a rate of 4°C/minute and then held for 2 minutes.

## Abbreviations

FAME: fatty acid methyl esters; GC-MS: gas chromatography-mass spectroscopy; NMR: nuclear magnetic resonance; O.D.: optical density; OMP: optimised *M. pulcherrima*; SMP: standard *M. pulcherrima*; YMD: yeast extract, malt extract and dextrose.

## Competing interests

A patent (UK No. 1302197.7) covering the majority of this work has been applied for. FS, RJS, CJC are the contributing authors.

## Authors’ contributions

FS carried out the majority of the culturing including the open-tank reactor experiments. FW carried out a few of the culturing experiments and handled the extracting and analysis of the products produced. RS and CC conceived of the study, participated in its design and coordination and drafted the manuscript. All authors read and approved the final manuscript.
