# Correction: Low-cost lipid production by an oleaginous yeast cultured in non-sterile conditions using model waste resources

**DOI:** 10.1186/1754-6834-7-42

**Published:** 2014-03-22

**Authors:** Fabio Santomauro, Fraeya M Whiffin, Rod J Scott, Christopher J Chuck

**Affiliations:** 1 Department of Biology and Biochemistry, University of Bath, Bath BA2 7AY, UK; 2 Centre for Sustainable Chemical Technologies, Department of Chemical Engineering, University of Bath, Bath BA2 7AY, UK

## Correction

After publication of this work [1], we noted that one of the authors was incorrectly named. The author list for this article should read Fabio Santomauro, Fraeya M. Whiffin, Rod J. Scott and Christopher J. Chuck
